# Acute-onset type 1 diabetes in an older patient with severe hypothermia and diabetic ketoacidosis (pH 6.84) triggered by traumatic hemothorax

**DOI:** 10.1530/EDM-25-0099

**Published:** 2026-01-30

**Authors:** Hono Kawashima, Mayu Watanabe, Momoka Hasegawa, Akihiro Katayama, Tomoko Yamanoi, Masaya Takeda, Satoshi Nogami, Kazuyuki Hida

**Affiliations:** ^1^ Department of Diabetology and Endocrinology, NHO Okayama Medical Center, Okayama, Japan; ^2^ Department of Anesthesiology, NHO Okayama Medical Center, Okayama, Japan

**Keywords:** diabetic ketoacidosis (DKA), hypothermia, type 1 diabetes mellitus, carbohydrate antigen 19-9 (CA19-9)

## Abstract

**Summary:**

A 77-year-old Japanese woman with a history of spinocerebellar degeneration, recently diagnosed with mild glucose intolerance managed by diet, sustained blunt chest trauma from a fall 7 days before admission. Three days prior, she developed general fatigue, thirst, and polydipsia. On arrival, she was unconscious, with unmeasurable blood pressure; heart rate of 112 beats/minute; and rectal temperature of 28°C. She presented with glucose levels of 1,125 mg/dL; β-hydroxybutyrate of 13,778 μmol/L; and arterial blood pH, 6.84, indicating severe diabetic ketoacidosis (DKA), likely triggered by traumatic hemothorax, and exhibited markedly elevated CA19-9 levels. External rewarming therapy was initiated along with vasopressor support, fluid resuscitation, continuous intravenous insulin infusion, and bicarbonate administration; subsequently, she regained consciousness. C-peptide depletion and positive islet-associated autoantibodies confirmed acute-onset type 1 diabetes mellitus. Although the CA19-9 level gradually decreased during hospitalization, it remained persistently above the reference range. Comprehensive imaging studies revealed no malignancy. Elevated CA19-9 levels were attributable to lung injury associated with traumatic hemothorax and multiple hepatic cysts. On hospital day 5, she was transferred to a general ward and resumed oral intake. Given the sustained improvement in glycemic control, she was transferred to another facility on hospital day 32. We report a rare case of acute-onset type 1 diabetes in an older patient who survived severe hypothermia and DKA secondary to traumatic hemothorax through intensive medical management. Appropriate and timely treatment can lead to favorable outcomes even in older patients with severe DKA and hypothermia. Furthermore, type 1 diabetes should be considered in elderly individuals presenting with newly developed glucose intolerance.

**Learning points:**

- Acute-onset type 1 diabetes mellitus can occur in older patients and may present with life-threatening complications, such as severe diabetic ketoacidosis (DKA) and hypothermia.
- Traumatic events, such as hemothorax from blunt chest trauma, can precipitate DKA even in patients with previously mild glucose intolerance.
- Intensive supportive care, including rewarming, insulin infusion, fluid resuscitation, vasopressors, and bicarbonate, can lead to recovery without the need for invasive interventions, such as CHDF or ECLS.
- Markedly elevated CA19-9 levels in the absence of malignancy may result from non-neoplastic conditions, such as lung injury and hepatic cysts, emphasizing the importance of thorough differential diagnosis.
- Positive islet autoantibodies and depleted C-peptide levels confirmed the diagnosis of acute-onset type 1 diabetes, reinforcing the need to consider autoimmune diabetes even in older patients with new-onset hyperglycemia.

## Background

Older patients with severe diabetic ketoacidosis (DKA) have a significantly high mortality risk (1, 2), which increases further when complicated by hypothermia (core body temperature <35°C) (3). In addition, elderly-onset diabetes and hyperglycemia may be an initial manifestation of malignancies, such as pancreatic cancer, necessitating careful differential diagnosis in such cases (4).

We encountered a case of a 77-year-old patient who developed both severe hypothermia and DKA following an incidental traumatic injury. The patient was successfully treated with intensive supportive care in the intensive care unit (ICU), without using invasive treatments such as extracorporeal life support (ECLS) or continuous hemodiafiltration (CHDF). During admission, the patient’s carbohydrate antigen (CA19-9) levels were markedly elevated, prompting further investigations to exclude malignancy. However, the patient was ultimately diagnosed with acute-onset type 1 diabetes mellitus (T1DM).

This case suggests that appropriate initial treatment can improve prognosis even in older patients with severe DKA and hypothermia, underscoring the importance of carefully examining older patients with newly developed glucose intolerance, considering the possibility of T1DM.

## Case presentation

A 77-year-old Japanese woman with a history of hypertension, dyslipidemia, osteoporosis, and subclinical hypothyroidism had been receiving treatment at another hospital. She was previously diagnosed with spinocerebellar degeneration at age 66. She exhibited dysarthria, truncal ataxia with gait instability, and intention tremor of the limbs, with no family history of the disease. Genetic testing for spinocerebellar ataxia had not been performed. She maintained independence in activities of daily living. She had a history of lung cancer surgery; however, she had no personal history of autoimmune disease. Her regular medications included celecoxib 5 mg, pravastatin 10 mg, risedronate 17.5 mg, limaprost alfadex 5 μg, suvorexant 10 mg, and azilsartan 20 mg. She had no family history of diabetes, and her sister was diagnosed with polycystic liver disease.

Three months before presentation, mild impaired glucose tolerance was first identified, and she was managed with dietary therapy alone. Seven days before admission, the patient fell at home, sustaining blunt chest trauma. Three days before admission, she experienced general fatigue, thirst, and polydipsia. Her family last confirmed her well-being on the night before admission. On the morning of admission, she was found collapsed in the living room and was urgently transported to our hospital due to impaired consciousness.

On arrival, she was unconscious (Japan Coma Scale III-300), with an unmeasurable blood pressure, a heart rate of 112 beats per minute, and a rectal temperature of 28°C. Pupillary light reflexes were diminished; pupils measured 4 mm bilaterally. Her face was pale, and black vomit was noted on her clothing. Lung sounds were clear, and her abdomen was flat and soft; however, her extremities were markedly cold. Extensive subcutaneous hemorrhage was observed in her right upper arm. Her height was 143.8 cm, weight 37.9 kg, and body mass index 18.3 kg/m^2^.

## Investigation

**Figure 1:**
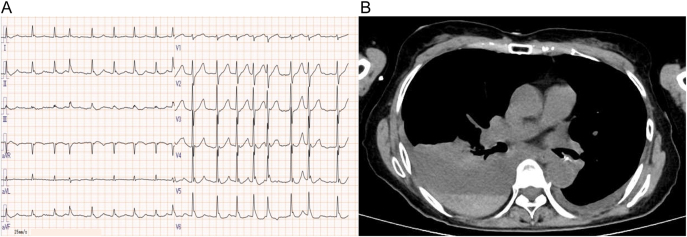
Examination results on emergency admission. (A) Electrocardiogram at presentation demonstrating an irregular rhythm and prominent J-waves in leads I, II, aVR, aVF, and V1–V6. (B) Chest computed tomography at presentation showed a right pleural effusion with partial high intensity.

**Figure 2:**
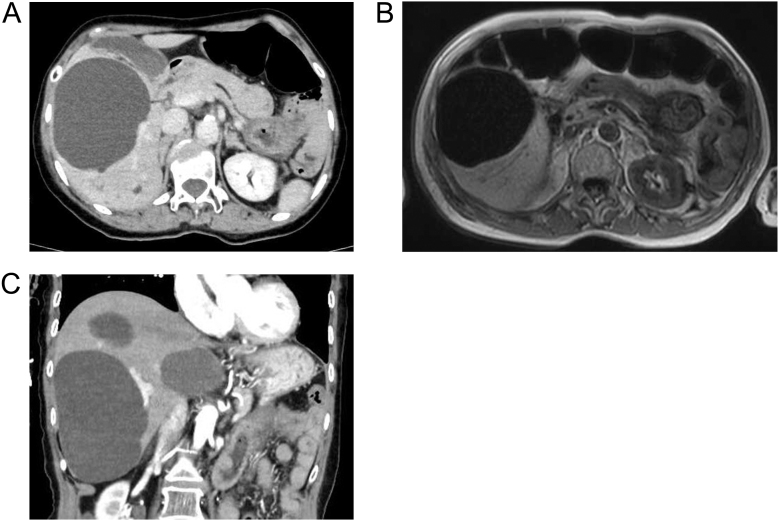
Imaging findings after hospitalization. (A) Dynamic contrast-enhanced CT of the pancreas showed no significant abnormalities. (B) MRI image of the pancreas showed no significant abnormalities. (C) Dynamic contrast-enhanced CT of the liver showed multiple hepatic cysts.

Laboratory findings at admission revealed HbA1c levels of 11.1% and a glucose level of 1,125 mg/dL. Arterial blood gas analysis showed a pH of 6.84, HCO_3_^−^ of 3.5 mmol/L, and an anion gap of 30.5 mmol/L, indicating marked hyperglycemia and high-anion gap metabolic acidosis. β-hydroxybutyrate was also elevated, leading to DKA diagnosis. She presented with acute kidney injury due to dehydration, with elevated pancreatic enzymes, amylase 225 U/L and lipase 226 U/L, and markedly elevated CA19-9 levels, 4,158 U/mL (Table 1). Endocrinologic evaluation showed thyroid-stimulating hormone at 1.2 µIU/mL, thyroxine at 1.32 ng/dL, cortisol at 93.9 μg/dL, and adrenocorticotropic hormone at 156.2 pg/mL, all within reference limits. Both anti-thyroid peroxidase and anti-thyroglobulin antibodies were negative. Owing to marked elevation of the pancreatic enzymes, anti-mitochondrial antibody was also tested but was negative. The electrocardiogram (ECG) at presentation showed an irregular heart rate of 101 bpm, with Osborn (J) waves present in leads I, II, aVR, aVF, and V1–V6 (Fig. 1A). Echocardiography showed mild mitral regurgitation but no obvious wall motion abnormalities, with relatively preserved left ventricular ejection fraction (50%). Computed tomography (CT) of the head showed no obvious intracranial hemorrhage, and chest CT showed a right 5th–7th rib fracture and a right pleural effusion with hyperabsorbent areas, leading to a diagnosis of multiple rib fractures and traumatic hemothorax (Fig. 1B). Accordingly, dynamic CT imaging of the pancreas, magnetic resonance cholangiopancreatography, and abdominal ultrasonography were performed; no pancreatic mass lesion suggestive of malignancy was identified (Fig. 2A and B).

**Table 1:** Patient’s laboratory data on admission.

Laboratory tests	Values
CBC	
WBC, /μL	13,400
Neutrophil, %	87.1
Lymphocytes, %	8
Monocytes, %	4.5
Eosinophils, %	0.1
Basophils, %	0.3
RBC, /μL	425 × 10⁴
Hemoglobin, g/dL	13.1
Platelets, /μL	32.8 × 10⁴
Urine	
Protein	±
Glucose	4＋
Ketone	－
Biochemical tests	
Total protein, g/dL	6.8
Albumin, g/dL	4.2
AST, U/L	18
ALT, U/L	22
LDH, U/L	321
ALP, U/L	108
γGTP, U/L	54
Creatinine	2.64
Urea nitrogen, mg/dL	79
Ammonia, μg/dL	114
Amylase, U/L	225
Lipase, U/L	226
Sodium, mg/dL	134
Potassium, mg/dL	5.1
Chloride, mg/dL	91
C-reactive protein, mg/dL	0.18
CA19-9, U/mL	4,158.8
Ketone body	
Total ketone body, μmol/L	14,311
Acetoacetate, μmol/L	533
β-hydroxybutyrate, μmol/L	13,778
Arterial blood gas analysis	
pH	6.841
PaCO₂, mmHg	20.4
PaO₂, mmHg	363
Bicarbonate, mmol/L	3.5
Base excess, mmol/L	−29.4
Anion gap, mmol/L	30.5
Diabetic endocrine factors	
Blood glucose, mg/dL	1,125
HbA1c, %	11.1
CPR, ng/mL	3.04

AST, aspartate aminotransferase; ALT, alanine aminotransferase; LDH, lactate dehydrogenase; ALP, alkaline phosphatase; γGTP, γ-glutamyl transpeptidase; CA19-9, carbohydrate antigen 19-9; paCO_2_, partial pressure of carbon dioxide; paO_2_, partial pressure of oxygen; HbA1c, glycated hemoglobin; CPR, C-peptide immunoreactivity.

## Treatment

**Figure 3:**
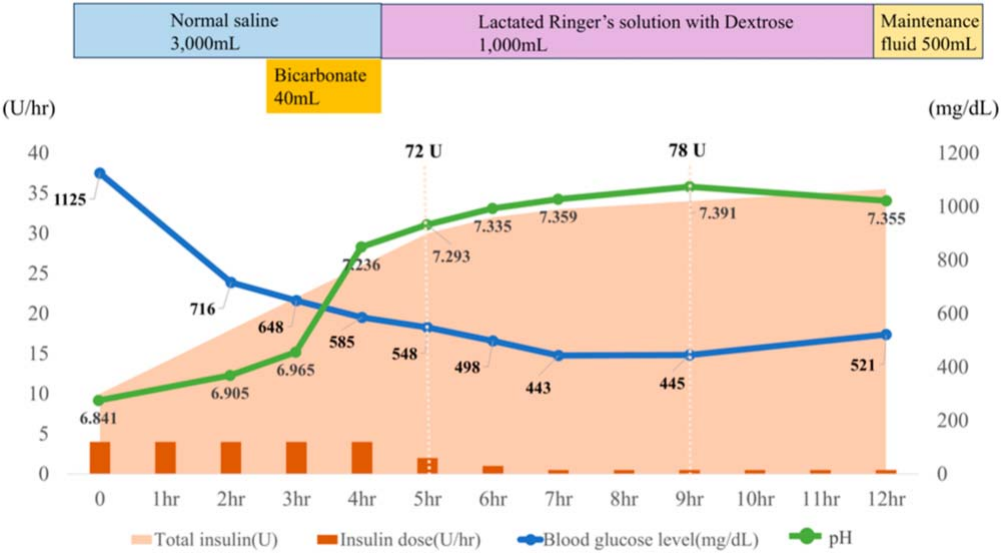
Time course after ICU admission illustrates the clinical response to treatment, showing intravenous fluid volume, insulin dose (U/h), blood glucose level (mg/dL), and arterial pH values.

At the ICU, external rewarming therapy was initiated along with vasopressor support (norepinephrine: 0.13–0.31 μg/kg/min; vasopressin: 0.008–0.033 U/min), fluid resuscitation, continuous intravenous insulin infusion (4 units/h), and sodium bicarbonate administration. CHDF was considered but not initiated, as the patient had strongly declined dialysis therapy in the past, and her family wished to honor that decision. As initial fluid resuscitation, isotonic saline was administered at a rate of 1,000 mL/h for 2 h, followed by 500 mL/h for 1 h and then 200–300 mL/h for 5 h, with a total volume exceeding 3,000 mL over 5 h. Insulin infusion was started at a rate of 0.1 units/kg/h, targeting a glucose reduction of 50–70 mg/dL per hour. Owing to insufficient glycemic response, 10-unit boluses of regular insulin were administered at initiation, for a total insulin dose of 72 units during the first 5 h, resulting in improved acidosis, with arterial pH increasing to 7.293 about 5 h after treatment initiation. Hemodynamics stabilized, glucose level decreased to 548 mg/dL (Fig. 3), and she regained consciousness and was able to engage in conversation. Her rectal temperature rose to 35°C after 7 h of external rewarming therapy, and the Osborn waves (J waves) on the ECG disappeared. Electrolyte abnormalities were corrected with potassium chloride and sodium phosphate supplementation and remained within reference ranges thereafter. On the third hospital day, due to progressive anemia with a hemoglobin level of 7.4 g/dL, a blood transfusion was administered. Upper gastrointestinal endoscopy subsequently revealed a duodenal ulcer. The anemia was attributed to both bleeding from the ulcer and traumatic hemothorax. Following the addition of a proton pump inhibitor, her hemoglobin levels improved. However, she developed marked dyspnea secondary to right pleural effusion associated with the traumatic hemothorax. On the fourth hospital day, thoracentesis was performed, yielding 800 mL pleural fluid. Subsequently, her dyspnea resolved, and her condition improved with conservative management. She was transferred to the general ward on the fifth hospital day.

## Outcome and follow-up

Her serum CA19-9 levels were markedly elevated at 4,158 U/mL on admission. Comparison with prior imaging studies demonstrated a tendency for size progression. No findings suggestive of cyst infection, compression of surrounding organs, or contrast defects in the superior or inferior mesenteric arteries were observed (Fig. 2C). CA19-9 levels gradually decreased over the clinical course, reaching 262 U/mL before discharge.

On hospital day 2, her glucose level was 351 mg/dL, and serum C-peptide immunoreactivity (CPR) was 0.17 ng/mL, indicating depleted insulin secretion. During the clinical course, positivity for both anti-GAD antibody (23.8 U/mL) and anti-IA-2 antibody (6.6 U/mL) was identified, leading to a diagnosis of acute-onset T1DM. Oral intake was initiated on hospital day 9; her pre-prandial blood glucose levels stabilized within the range of 100–200 mg/dL with a total daily insulin dose (TDD) of 10 units. A glucagon stimulation test performed on hospital day 22 showed a ΔCPR of 0.65 ng/mL (from 1.04 ng/mL at baseline to 1.69 ng/mL at 6 min), indicating a slight improvement in endogenous insulin secretion. Furthermore, the CPR index improved to 1.48 on hospital day 28; however, insulin therapy was continued.

## Discussion

We encountered a case of acute-onset T1DM in an older patient who presented with severe hypothermia and DKA, triggered by traumatic hemothorax, and exhibited markedly elevated CA19-9 levels.

Trauma-induced DKA is extremely rare. Previous reports have primarily described cases in which antidiabetic medications contributed to the development of DKA following traumatic brain injury (5, 6) and a case of severe hemothorax due to cardiac perforation following percutaneous coronary intervention that was complicated by metformin-associated lactic acidosis (7). In contrast, our patient developed severe DKA and hypothermia without prior diabetes treatment, and the hemothorax may have further worsened acidosis due to reduced tissue oxygen delivery in hemorrhagic shock (8, 9), highlighting trauma as an independent trigger.

She presented profound metabolic acidosis (pH: 6.84, β-hydroxybutyrate: 13,778 μmol/L, and bicarbonate: 3.5 mmol/L) and severe hypothermia (rectal temperature of 28°C) (10, 11). Hypotension with a systolic blood pressure <90 mmHg, a risk factor for imminent cardiac arrest, led to consideration of ECLS (12). The outdoor temperature was −0.7°C, but the indoor environment was not cold. In DKA, insulin deficiency impairs cellular glucose uptake, reducing heat production and causing hypothermia that exacerbates metabolic derangements (13). In this patient, the absence of environmental triggers suggests that DKA itself precipitated and worsened the hypothermia. Prompt rewarming to >30°C usually prevents neurological sequelae in DKA-associated hypothermia (3, 14). The rectal temperature increased to 35°C within 7 h in this patient, and she recovered without neurological deficits.

This case was also notable due to the patient’s poor response to norepinephrine, requiring the addition of vasopressin. Vasopressin, used as a secondary agent in catecholamine-resistant or septic shock, effectively maintained organ perfusion in this case (15, 16, 17). As consent for CHDF could not be obtained, adjunctive sodium bicarbonate was administered; although generally not recommended due to risks of hypokalemia and cerebral edema (18), it may be considered in severe acidosis with a pH < 7.0 (10), as demonstrated in this case.

Serum CA19-9 elevation is observed in various malignancies, including pancreatic cancer, biliary tract cancer, hepatocellular carcinoma, gastric cancer, genitourinary cancers, and gynecological cancers (19); however, it also increases in benign diseases. In older patients, careful evaluation to exclude malignancy is particularly important. In this case, the markedly elevated CA19-9 levels were initially observed, likely due to pulmonary injury from multiple rib fractures, while persistently elevated levels reflected underlying autosomal dominant polycystic liver disease (20, 21, 22, 23, 24). This highlights the importance of distinguishing benign from malignant causes when interpreting CA19-9 in older adults.

The diagnostic criteria for acute-onset type 1 diabetes mellitus include the following: i) diabetes symptom onset followed by ketosis or ketoacidosis within approximately 3 months, ii) need for continuous insulin therapy from the early stage of diagnosis, iii) presence of pancreatic islet-related autoantibodies, and iv) a fasting serum CPR <0.6 ng/mL, even in the absence of detectable autoantibodies, indicating endogenous insulin deficiency (25). In this case, the patient developed DKA approximately 3 months after being identified with impaired glucose tolerance. Serum CPR was depleted at 0.17 ng/mL, and islet-associated autoantibodies were positive, leading to a diagnosis of acute-onset T1DM. Following diagnosis, patients may experience a partial remission phase, commonly referred to as the ‘honeymoon period’, characterized by transient improvement in glycemic control (26).

Adult-onset T1DM patients are at an increased risk of developing other autoimmune diseases, with approximately 30% exhibiting thyroid autoimmunity (27). This patient had normal thyroid function and negative thyroid autoantibodies; however, GAD and IA-2 antibodies were positive. IA-2 antibody positivity is rare among patients with elderly-onset T1DM (28). A Japanese series of 22 elderly-onset T1DM patients reported multiple autoantibody positivity, including IA-2 antibodies, supporting the diagnosis of T1DM (29). These findings highlight the significance of assessing diabetes-related autoantibodies for confirming autoimmune etiology even in older adults.

This case report describes a single patient, and therefore, its findings may not be generalizable to a broader population.

This case demonstrates acute-onset T1DM in an older patient with severe hypothermia and DKA triggered by traumatic hemothorax. The patient was successfully rescued without the use of ECLS or CHDF. Markedly elevated serum CA19-9 levels were observed, likely attributable to multiple hepatic cysts and pulmonary injury, necessitating thorough evaluation to exclude malignancy. Positive islet autoantibodies and severely reduced insulin secretion ultimately confirmed the diagnosis of acute-onset T1DM, emphasizing the need for careful assessment of autoimmune markers even in elderly patients.

## Declaration of interest

The authors declare that there is no conflict of interest that could be perceived as prejudicing the impartiality of the work reported.

## Funding

This work did not receive any specific grant from any funding agency in the public, commercial, or not-for-profit sector.

## Patient consent

Written informed consent for the publication of this report and accompanying clinical details and clinical images was obtained from the patient in accordance with the journal’s patient consent policy.

## Author contribution statement

HK wrote the original draft of the manuscript. MW wrote, reviewed, and edited the original draft of the manuscript. MH and TY were involved in data curation. AK performed data curation and supervised the study. MT, SN, and KH supervised the study.
